# Rituximab in primary podocytopathies: efficacy and safety in a retrospective cohort

**DOI:** 10.1590/2175-8239-JBN-2025-0120en

**Published:** 2025-09-26

**Authors:** Gabriel Teixeira Montezuma Sales, Danilo Euclides Fernandes, Gianna Mastroianni Kirsztajn

**Affiliations:** 1 Universidade Federal de São Paulo, Departamento de Medicina, São Paulo, SP, Brazil.

**Keywords:** Rituximab, Podocytopathies, Focal and Segmental Glomerulosclerosis, Minimal Change Disease

## Abstract

**Introduction:**

Podocytopathies are an important cause of nephrotic syndrome, and immunosuppression plays a pivotal role in disease management. The efficacy of biological agents such as rituximab (RTX), however, remains unclear, especially in adults. This study hypothesized that RTX is efficient and safe in the treatment of steroid-sensitive and steroid-resistant primary podocytopathies.

**Method:**

A retrospective cohort study was conducted based on medical records before the first infusion of RTX (T0) and 1 to 3 months (T1) and 3 to 6 months after infusion (T2). Patients had biopsy-proven podocytopathies and received at least 500 mg of RTX. Individuals with secondary glomerular diseases were excluded.

**Results:**

A total of 31 patients with a mean age at infusion of 32.9 years (SD 11.0) were included. At T2, remission was reached in 45.2%, with complete remission in 19.4%. Prior response to steroids was related to a better prognosis, with remission in up to 68.5% of these patients. Moreover, 20.0% of steroid-resistant patients reached adapted remission (≥ 35% proteinuria decrease + ≥ 20% serum albumin increase). Hypertension and previous use of calcineurin inhibitors were not predictors of clinical response. The most frequent adverse events were infection (12.9%) and rash (9.7%).

**Discussion:**

In conclusion, our results suggest that RTX is a useful therapeutic option not only for steroid-sensitive podocytopathies, but also in selected steroid-resistant cases, determining a proteinuria decrease that can contribute positively to the clinical management of such glomerular diseases. RTX was generally efficient and well tolerated in this adult cohort.

## Introduction

Glomerulopathies are the third most common etiology of end-stage kidney disease (ESKD) in Brazil (9%) and in the United States of America (15.5%)^1,2^. Among primary glomerulopathies in Brazil, minimal change disease (MCD) and focal and segmental glomerulosclerosis (FSGS) are the most frequent causes of nephrotic syndrome in children and adults, respectively. Both are characterized by podocyte damage, and are collectively referred to in this study as podocytopathies, as classified by others^3,4^.

Currently, the management of podocytopathies remains a challenge, as side effects can be life threatening and clinical response may not be as satisfactory as expected. This motivated and investigation on the role of monoclonal antibodies in podocytopathies, particularly rituximab (RTX), a chimeric molecule (mouse/human) that binds to the CD20 surface antigen and induces B lymphocyte apoptosis^5,6^.

Some clinical trials on glomerulopathies have already shown that RTX is effective in ANCA-associated vasculitis, membranous nephropathy, and cryoglobulinemia^7, 8, 9, 10^. Ruggenenti *et al.* published their prospective cohort results in 2014, demonstrating that one dose of 375 mg/m^2^ was safe and effective in decreasing steroid dose and reducing disease recurrence in patients who were in podocytopathy remission^11^. Pediatric randomized studies have shown that two to four doses of 375 mg/m^2^ weekly prolong remission and reduce cumulative doses of steroids^12,13^. Our study aims to evaluate RTX efficacy and safety among patients with steroid-sensitive or steroid-resistant primary podocytopathies.

## Methods

This is a single center retrospective cohort study. From January 1998 to January 2020, we analyzed medical records from the glomerulonephritis outpatient clinic at Universidade Federal de São Paulo, Brazil. We included individuals who were 1) ≥ 12 years old, 2) with a biopsy-proven podocytopathy (FSGS or MCD), and 3) who received at least one dose of RTX during a minimum follow-up of 6 months, prescribed at the discretion of the assisting nephrologist. This sample was selected by a non-random process based on data availability. Patients with pre-RTX proteinuria <0.5 g/24h or secondary glomerular diseases were excluded. The study protocol was approved by the Research Ethics Committee of Universidade Federal de São Paulo (permit 3 496 678) and was devised in compliance with the principles of the Declaration of Helsinki.

Clinical and laboratory data utilized to assess outcomes included 24-hour urinary protein (or protein-to-creatinine ratio, if not available) and serum albumin at three time points: up to three months before RTX first infusion (T0), 1 to 3 months post-infusion (T1), and 3 to 6 months post-infusion (T2). Symptoms possibly associated to RTX infusion were registered for the following 12 months. To more precisely establish an eventual association between observed symptoms and RTX administration, symptoms reported 12 months before infusion were also registered.

### Outcomes

We categorized three remission criteria: 1) complete remission: urinary protein ≤ 0.3 g/24h with eGFR up to 25% lower than T0; 2) partial remission: reduction of 24-hour urinary protein > 50% compared to T0, urinary protein ≤ 3.5 g/24 h, and eGFR up to 25% lower than renal function before RTX infusion; and 3) adapted remission: increase in serum albumin > 20% or > 0.5 g/dL compared to T0, reduction in 24-hour urinary protein > 35%, and eGFR up to 25% lower than T0. The inclusion of an alternative remission criterion was supported by findings from previous studies indicating favorable prognosis even in cases without classic remission criteria, particularly when accompanied by increases in serum albumin levels^14, 15, 16, 17^. This approach was primarily based on the GEMRITUX trial, which employed a post hoc composite endpoint defined as a reduction in proteinuria greater than 50% combined with an increase in serum albumin exceeding 30%^18^. We also evaluated the proportion of patients with a 24-hour urinary protein decrease > 35%. Those who did not meet any of the above criteria were considered refractory to treatment.

### Statistical Analysis

**Figure 1:**
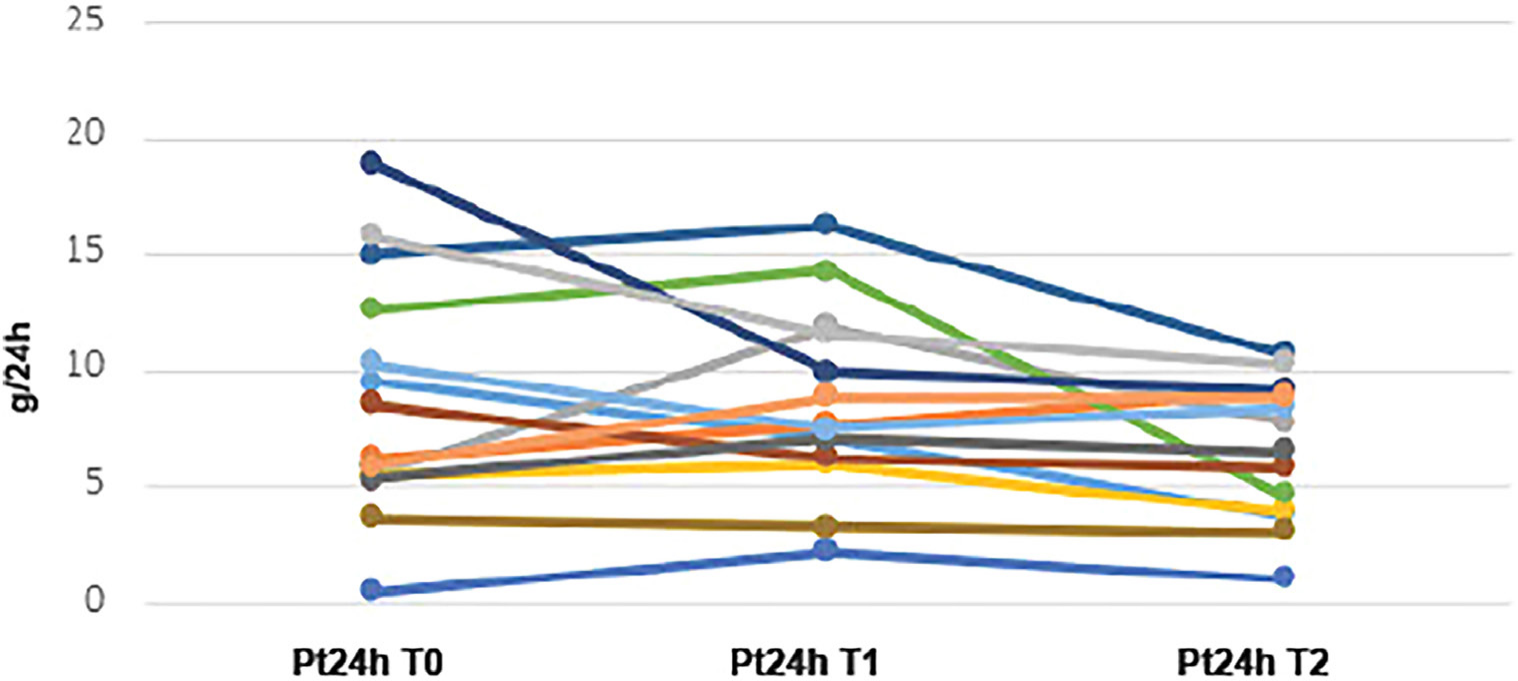
Evolution of 24-hour urinary protein at baseline (T0), T1, and T2 in patients with steroid-resistant podocytopathies.

**Figure 2.**
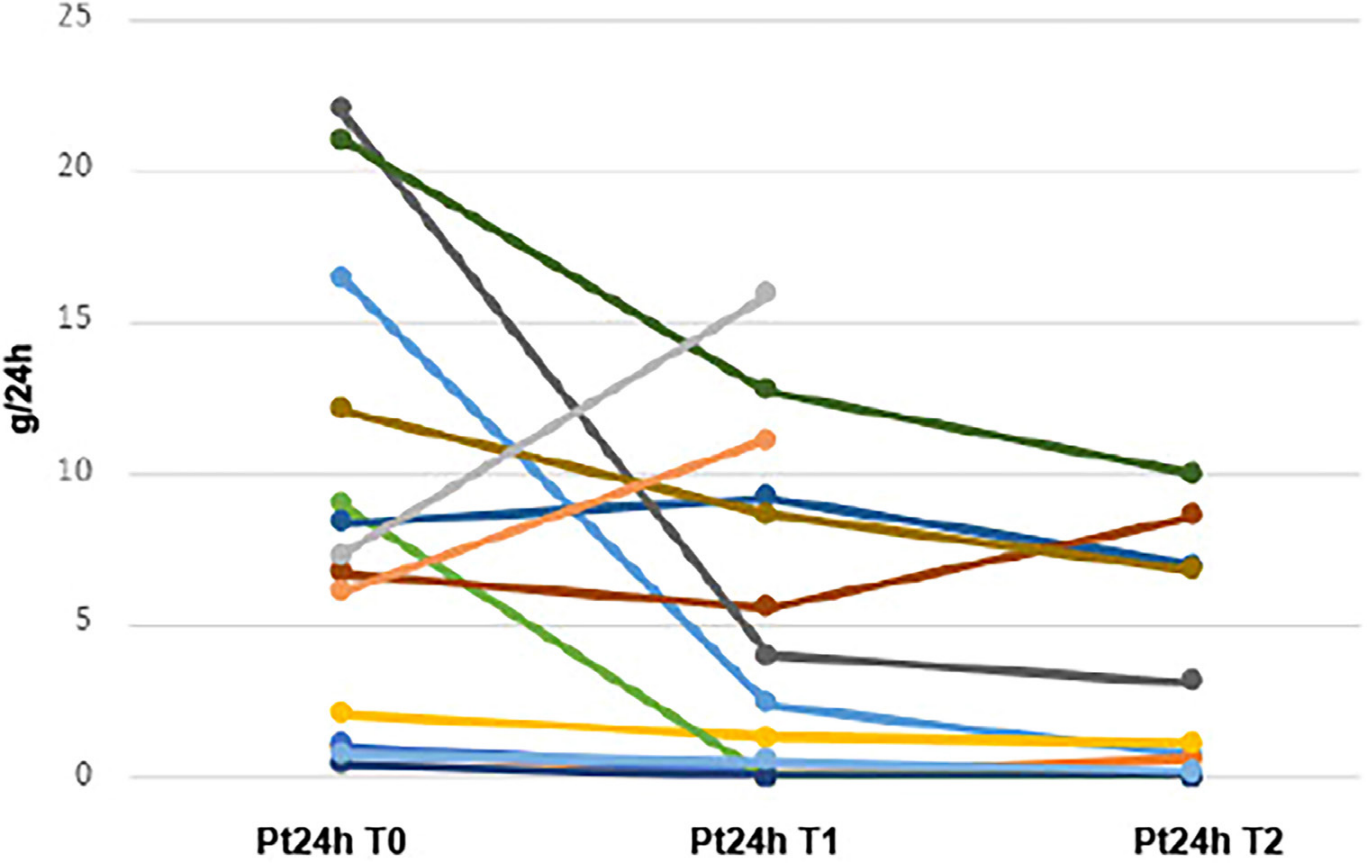
Evolution of 24-hour urinary protein at baseline (T0), T1, and T2 in patients with steroid-sensitive podocytopathies.

Data were collected from the medical records, organized in Microsoft Excel^®^, and summarized through absolute and relative frequencies (nominal variables) and mean and standard deviation (numeric variables). Line graphs were used to illustrate variations in proteinuria levels at time points T0, T1, and T2 (Figures 1 and 2).

Abbreviations – Pt24h: 24-hour urinary protein.

Abbreviations – Pt24h: 24-hour urinary protein.

## Results

Thirty-one patients with mean age of 32.9 (SD ± 11.0) years at RTX infusion and 24.8 (SD ± 13.4) years at first symptoms were evaluated, of whom 17 (54.8%) were men. Ten individuals (32.3%) presented their first symptoms before the age of 21 years and five individuals (16.1%) before the age of 13 years. At T0, their mean eGFR was 74.5 mL/min/1.73 m^2^ (SD ± 32.3), serum albumin 3.0 g/dL (SD ± 1.0), and urinary protein 7.9 g/24h (SD ± 6.2; 74.2% > 3.5 g/24h). About half of the participants (51.6%) were steroid-sensitive and 87.1% received another immunosuppressor before RTX, especially prednisone (35.5%), cyclosporine (19.4%), and cyclosporine and prednisone combined (16.1%). Most patients (90.3%) were also receiving renin-angiotensin-aldosterone system inhibitors (Table 1). At T1 (1 to 3 months after RTX infusion), 25.8% achieved one of the study outcomes – 23.3% adapted remission, 3.2% partial remission, and 16.1% complete remission. At T2 (3 to 6 months after RTX infusion), 41.9% achieved one of our outcomes – 33.3% adapted remission, 3.2% partial remission, and 19.4% complete remission.

**Table 1:** Baseline characteristics of the patients with podocytopathies treated with rituximab

Demographics	N = 31
Male, N (%)	17 (54.83)
Age, years (SD)	32.90 (11.04)
Age at first symptoms, years (SD)	24.80 (13.42)
**Biochemistry, mean (SD)**	
Cr mg/dL	1.34 (0.76)
eGFR ml/min/1.73m^2^	74.52 (32.27)
Urinary protein g/24h	7.95 (6.20)
Serum albumin g/dL	3.05 (1.00)
**Pre-Infusion Therapy, N (%)**	
Csa + prednisone Csa Prednisone RAASi Other No ISS	5 (16.13) 6 (19.36) 11 (35.48) 28 (90.32) 5 (16.13) 4 (12.90)
**Clinical Features**	
Hypertension, No (%)	21 (67.74)
RTX total dose mg/m^2^, mean (SD)	663.77 (234.56)
RTX 2 infusions, N (%)	26 (83.87)
Steroid-resistant, N (%)	15 (48.38)
**Histological pattern, N (%)**	
FSGS	22 (70.96)
MCD	9 (29.04)

Abbreviations – Cr: serum creatinine; Csa: cyclosporine; FSGS: focal segmental glomerulosclerosis; ISS: immunosuppressor; MCD: minimal change disease; RAASi: renin-angiotensin-aldosterone system inhibitor; RTX: rituximab.

**Figure 3.**
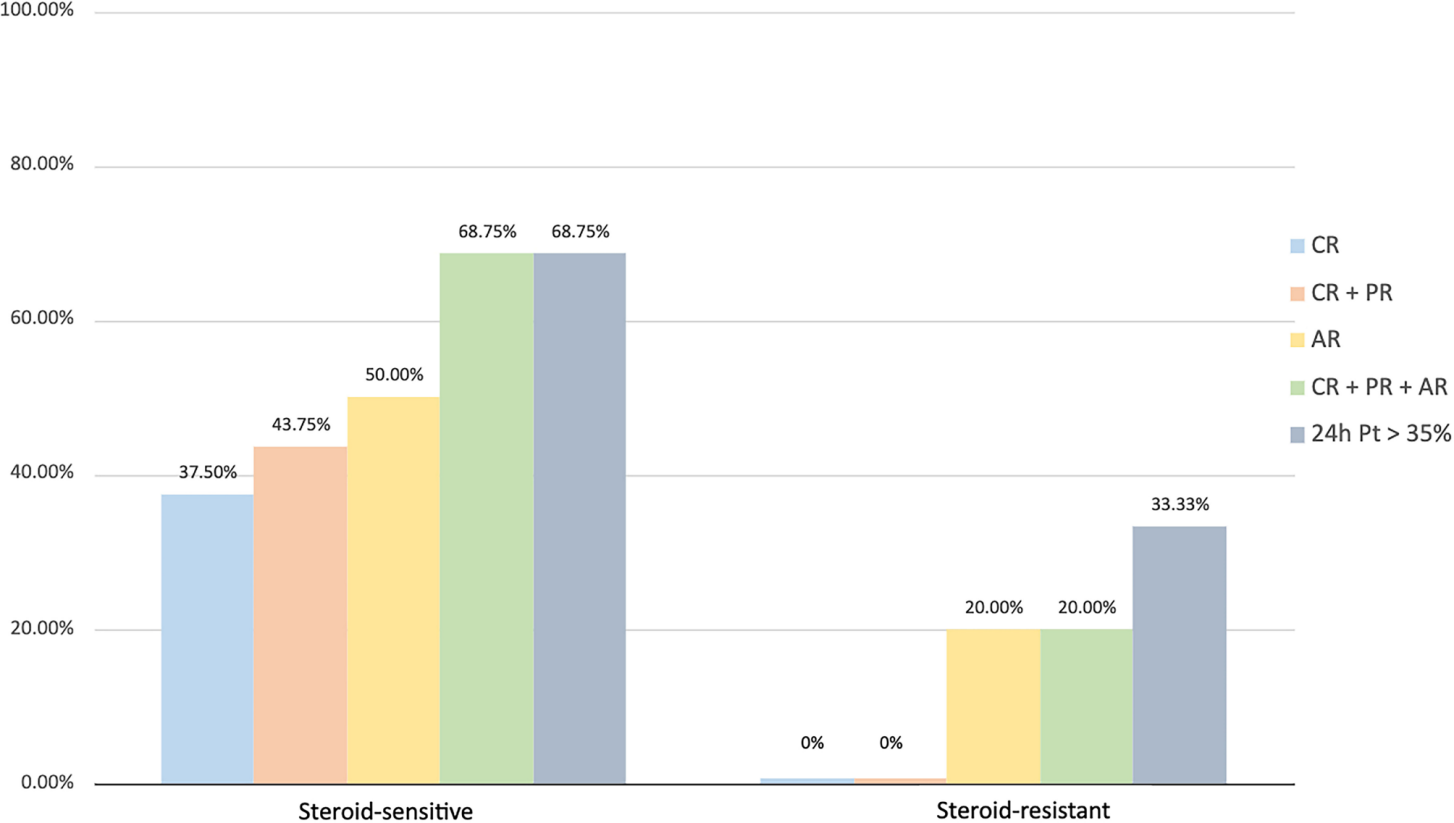
Distribution of complete, complete + partial remissions, adapted remission, and 24h urinary protein reduction >35% in T2 according to corticosteroid sensitivity.

Figure 3 shows the distribution of subjects according to the remission status and steroid sensitivity. Among steroid-sensitive patients, 68.7% presented any of the possible outcomes at T2 – 37.5%, complete remission and 50.0% adapted remission. Among steroid-resistant patients, complete and adapted remission rates at T2 were 0.0% and 20.0%, respectively.

Abbreviations – AR: Adapted Remission; CR: Complete Remission; PR: Partial Remission; 24h Pt > 35%, 24h urinary protein reduction > 35%.

Table 2 presents the characteristics of the individuals who did not respond to RTX (no remission group) and of those who reached: 1) complete or partial remission and 2) adapted remission. Considering only the relative frequencies and refraining from inferential statistical analysis due to the small sample and non-random selection, it was observed that non-responding patients received a lower cumulative dose of rituximab (643.2 – SD 216.4 vs 722.8 – SD 301.8), had prior exposure to cyclosporine (95.6% vs 37.5%), and exhibited higher CD19 cell counts following rituximab infusion (43.7 – SD 113.9 vs 3.16 – SD 5.6). They were also predominantly hypertensive (65.2%), steroid-resistant (65.2%), and presented with FSGS as the histological pattern (78.3%). In contrast, patients who achieved complete or partial remission after rituximab treatment had higher baseline serum albumin levels (3.6 – SD 1.0 vs 2.8 – SD 0.9), no diagnosis of hypertension (12.5%), and were steroid-sensitive (100%). Patients with adapted remission exhibited higher baseline proteinuria (12.7 – SD 7.6 vs 5.7 – SD 4.0), lower serum albumin levels (2.5 – SD 0.9 vs 3.3 – SD 0,9), and lower eGFR (63.5 – SD 34.7 vs 80.1 – SD 31.3).

**Table 2:** Clinical and laboratory characteristics of the 31 patients with podocytopathies according to presence and type of remission

	Complete or partial remission		Adapted remission	
Features	Remission (8)	No response (23)	Remission (10)	No response (20)
Age of first symptoms, years (SD)	23.75 (7.70)	25.18 (15.11)	23.44 (12.72)	24.05 (13.06)
Pre-infusion urinary protein g/24h (SD)	6.40 (8.56)	8.50 (5.29)	12.66 (7.60)	5.68 (4.02)
Pre-infusion serum albumin g/dL (SD)	3.63 (1.04)	2.84 (0.92)	2.46 (0.90)	3.34 (0.93)
eGFR mL/min/1.73m^2^ (SD)	77.52 (42.92)	73.47 (28.80)	63.50 (34.67)	80.14 (31.26)
RTX total dose mg/m^2^ (SD)	722.81 (301.85)	643.23 (216.40)	693.03 (280.55)	654.10 (226.76)
CD19 cell/mm^3^ (SD), n = 11	3.16 (5.60)	43.72 (113.90)	3.43 (5.42)	50.82 (123.06)
Pre-infusion cyclosporine, N (%)	3 (37.50)	22 (95.65)	8 (80.00)	16 (80.00)
Hypertension, N (%)	1 (12.50)	15 (65.22)	4 (40.00)	11 (55.00)
FSGS, N (%)	4 (50.00)	18 (78.26)	9 (90.00)	13 (65.00)
MCD, N (%)	4 (50.00)	5 (21.74)	1 (10.00)	7 (35.00)
Steroid-sensitive, N (%)	8 (100)	8 (34.78)	7 (70.00)	9 (45.00)

Abbreviations – eGFR: Estimated Glomerular Filtration Rate; MCD: Minimal Change Disease; FSGS: Focal and Segmental Glomerulosclerosis; RTX: Rituximab.

Table 3 shows self-reported symptoms 1 year before and after RTX. Up to 12 months after RTX infusion, most subjects reported no adverse events (61.3%), followed by rash (9.7%), arthralgia (6.5%), and pneumonia (6.5%). There was no increase in adverse events after RTX infusion in comparison with 12 months before RTX. No deaths were registered and one of the patients progressed to ESKD within 12 months (a 22-year-old male patient, who first visited a nephrologist a year before his first RTX infusion due to a steroid resistant FSGS).

**Table 3:** Self-reported adverse events up to 1-year before and after rituximab infusion

Adverse events	Before, N	%	After, N	%
Urinary tract infection	4	12.90%	1	3.23%
Arthralgia	4	12.90%	2	6.45%
Viral infection	3	9.68%	1	3.23%
Skin infection	2	6.45%	0	0%
Pneumonia	2	6.45%	2	6.45%
Rash	0	0%	3	9.68%
Other	5	16.13%	4	12.90%
None	13	41.94%	19	61.30%

Line graphs (Figures 1 and 2) depict the progression of proteinuria after RTX administration at time points T0, T1, and T2, which suggest that reductions in proteinuria were more significant and occurred with greater frequency in steroid-sensitive individuals compared to steroid-resistant individuals, although a reduction in proteinuria can also be observed in some steroid-resistant cases.

## Discussion

This retrospective cohort aimed to evaluate efficacy and safety of RTX according to medical records of 31 patients who had biopsy-proven primary podocytopathy. Also, we explored some clinical features that may have an impact on complete and partial remission.

Novel and better options for podocytopathies treatment are urgently needed given that patients with FSGS present a remission rate of nearly 50% and a risk of progressing to ESKD of up to 20% within a decade. Furthermore, these individuals are exposed to substantial lifetime cumulative steroid doses, leading to a significant risk of adverse events^19, 20, 21^. RTX has already been suggested to be efficient in other glomerulopathies, such as membranous nephropathy and ANCA-associated vasculitis, but strong evidence of efficacy in podocytopathies is still lacking^7, 8, 9, 10,22^.

Our cohort showed lower remission rates than reported by others, possibly because approximately half of our patients (48.4%) were steroid-resistant. A 2020 meta-analysis showed a remission (partial and complete) rate of 73.7%, but most studies focused on steroid-sensitive patients. When considering only steroid-sensitive patients, 50.0% achieved partial or complete remission after RTX, consistent with the findings of Hansrivijit *et al*.^23^.

Although RTX dose was not associated with clinical remission, Chan *et al.*, in a retrospective cohort, found higher recurrence rates in 511 children who used a total dose of 375 mg/m^2^ when compared to > 750 mg/m^2 24^. On the other hand, Ruggenenti *et al.*, in 2014, found that 375 mg/m^2^ was highly efficient in preventing recurrence in patients with FSGS or MCD in remission^11^.

As an antibody, RTX can be excreted in urine of patients with nephrotic syndrome, and it is traditionally recommended that RTX be used after remission induction in podocytopathies. It is of note that most of our patients had nephrotic syndrome at T0, but we still observed a reasonable remission rate when considering steroid-sensitive patients, using a mean total RTX dose of 663.7 mg/m^2^. Patients who did not respond received lower doses of rituximab on average, although the absence of inferential analysis prevents drawing stronger conclusions. The lack of randomized trials in adults complicates recommendations of a specific posology in podocytopathies, but steroid responsiveness, remission status, and histological pattern should be considered before choosing RTX dose^24, 25, 26, 27^.

Hypertension and prior use of cyclosporine were associated with lack of response to RTX, probably reflecting more refractory disease. FSGS histological pattern was also a risk factor for no response, with only 18.2% (4 from 22 patients) showing partial or complete remission, while 44.4% of MCD patients reached remission. The same was observed by other groups, as demonstrated by Hansriviji *et al.* who found partial and complete remission rates of 80.3% in MCD and 53.6% in FSGS^23^.

Although FSGS subtypes – primary, genetic, and secondary – share clinical characteristics like nephrotic proteinuria, they differ in their physiopathology and cannot usually be distinguished by histology^28,29^. These differences may influence response rates, with non-immune mechanisms potentially more prevalent in the steroid-resistant group. Interestingly, five steroid-resistant patients (33.3%) presented a > 35% reduction in proteinuria, possibly due to a non-immune-mediated effect. In addition to its well-known effect on B cells, RTX stabilizes podocytes’ cytoskeleton by regulating acid sphingomyelinase and connecting to acid-similar-3b sphingomyelinase, both of which may reduce proteinuria^30,31^. This may explain why 20.0% of the patients in the steroid-resistant group presented adapted remission.

The role of RTX in steroid-resistant podocytopathies remains unclear. In our cohort, none of the fifteen steroid-resistant patients presented complete or partial remission, but 3 (20.0%) presented adapted remission and 5 (33.3%) a reduction higher than 35% in 24 h urinary proteinuria, with a relatively high dose of RTX. A non-randomized and non-controlled study with 9 patients conducted at the Mayo Clinic showed no statistical significance in reducing proteinuria after six months (5.9 ± 4.6 g/24h) and after 12 months (7.7 ± 4.6 *vs* 7.2 ± 7.3 g/24h)^32^. On the other hand, a systematic review including 226 children with steroid-resistant nephrotic syndrome showed a response rate of 39.2% in FSGS^33^. Considering the heterogeneous evidence, the 2021 KDIGO guidelines for glomerulopathies place RTX as a therapeutic option only in steroid-sensitive patients, but emphasizes the need for further high-impact studies for a definitive response^22^.

We also observed lower prevalence of adverse events (infections, 12.9%; rash, 9.7%; arthralgia, 6.5%) in our cohort than in other studies, a finding that may have been affected by a lower rate of combined RTX and other immunosuppressants. In a retrospective cohort of 468 patients ≥ 21 years old, the main adverse events up to 24 months after RTX infusion were infections (47.9%), part of which were considered severe (37.5%). Similarly, IgM hypogammaglobulinemia was found in 40.8%, 19.1% had anaphylaxis, and 19.0% of the patients had infusion reactions – hypotension (3.4%), respiratory symptoms (3.0%), tremors (3.0%) and rash or itching (2.6%)^34^. In another study involving 2578 rheumatoid arthritis patients, a 25% infusion reaction rate was observed; however, only 1% of these reactions were classified as severe^35^.

As a chimeric antibody, RTX may induce infusion-related reactions. Given the retrospective nature of our study, mild symptoms may have been suppressed, which would explain the low incidence of infusion-related reactions. On the other hand, the lack of documentation suggests that unreported events were likely mild.

Strengths of this study include 1) it is one of the largest cohorts with RTX in podocytopathies; 2) it offers new insights by suggesting that pre-infusion serum albumin and proteinuria, in addition to hypertension and steroid-responsiveness, may predict clinical response to RTX; 3) it demonstrates proteinuria decrease in some steroid-resistant patients, supporting the hypothesis that RTX may have some effect in these patients; 4) most of our patients had nephrotic syndrome in T0, challenging the common belief that RTX should be used during remission to diminish urinary losses.

Some of the limitations of our study are 1) its retrospective design is affected by memory bias in self-reported symptoms and data are restricted to medical records; 2) its small sample size, precluding an inferential analysis; 3) different regimens of RTX infusion, which leads to unstandardized doses and intervals; 4) concomitant use of other immunosuppressive drugs, making it difficult to attribute observed effects solely to RTX; 5) absence of genetic tests, for better definition of FSGS subtypes.

In conclusion, our results suggest that RTX is a useful therapeutic option not only for steroid-sensitive podocytopathies, but also in selected steroid-resistant cases, determining a proteinuria decrease that can contribute positively to the clinical management of such glomerular diseases. RTX was generally efficient and well tolerated in this adult cohort.

## Data Availability

The data underlying this article will be shared on reasonable request to the corresponding author.
