# Supplementary material for: Rituximab in primary podocytopathies: efficacy and safety in a retrospective cohort
Source: J Bras Nefrol. 2025 Sep 26;47(4):e20250120. doi: 10.1590/2175-8239-JBN-2025-0120en (PMC12477824; doi:10.1590/2175-8239-JBN-2025-0120en)
Supplement: Translation

# Translation: Rituximabe em podocitopatias primárias: eficácia e segurança em uma coorte retrospectiva

**DOI:** 10.1590/2175-8239-JBN-2025-0120pt

**Authors:** Gabriel Teixeira Montezuma Sales, Danilo Euclides Fernandes, Gianna Mastroianni Kirsztajn

**Affiliations:** 1 Universidade Federal de São Paulo, Departamento de Medicina, São Paulo, SP, Brasil.

**Keywords:** Rituximabe, Podocitopatias, Glomerulosclerose Segmentar e Focal, Doença de Lesão Mínima

## Abstract

**Introdução:**

Podocitopatias constituem uma causa importante de síndrome nefrótica. A imunossupressão desempenha papel fundamental no manejo da doença. Entretanto, a eficácia de agentes biológicos, como o rituximabe (RTX), permanece incerta, especialmente em adultos. Este estudo postulou que o RTX é seguro e eficaz no tratamento de podocitopatias primárias sensíveis, resistentes a esteroides.

**Método:**

Estudo de coorte retrospectivo baseado em registros médicos anteriores à primeira infusão de RTX (T0), 1 a 3 meses (T1) e 3 a 6 meses após a infusão (T2). Os pacientes apresentaram podocitopatias comprovadas por biópsia e receberam pelo menos 500 mg de RTX. Excluíram-se indivíduos com doenças glomerulares secundárias.

**Resultados:**

Foram incluídos 31 pacientes com idade média à infusão de 32,9 anos (DP 11,0). No T2, a remissão foi alcançada em 45,2% (remissão completa em 19,4%). A resposta prévia aos corticosteroides associou-se a melhor prognóstico, com remissão em até 68,5% desses pacientes. Adicionalmente, 20,0% dos pacientes resistentes aos esteroides alcançaram remissão adaptada (≥ 35% de redução da proteinúria + ≥ 20% de aumento da albumina sérica). Hipertensão e uso prévio de inibidores da calcineurina não foram preditores de resposta clínica. Os eventos adversos mais frequentes foram infecção (12,9%) e exantema (9,7%).

**Discussão:**

Em conclusão, nossos resultados sugerem que RTX é uma opção terapêutica útil não apenas para podocitopatias sensíveis a esteroides, mas também em casos selecionados resistentes a esteroides, determinando uma redução da proteinúria que pode contribuir positivamente para o manejo clínico dessas doenças glomerulares. RTX foi, em geral, eficaz e bem tolerado nesta coorte adulta.

## Introdução

As glomerulopatias constituem a terceira etiologia mais comum de doença renal em estágio V (DRCVD) no Brasil (9%) e nos Estados Unidos (15,5%)^1,2^. Entre as glomerulopatias primárias no Brasil, a doença de lesão mínima (DLM) e a glomeruloesclerose segmentar e focal (GESF) são as causas mais frequentes de síndrome nefrótica em crianças e adultos, respectivamente. Ambas são caracterizadas por lesão podocitária e são referidas coletivamente neste estudo como podocitopatias, conforme classificação adotada por outros autores^3,4^.

Atualmente, o manejo das podocitopatias continua sendo um desafio, uma vez que os efeitos adversos podem oferecer risco à vida e a resposta clínica pode não ser tão satisfatória quanto o esperado. Isso motivou uma investigação sobre o papel dos anticorpos monoclonais nas podocitopatias, especialmente o rituximabe (RTX), uma molécula quimérica (camundongo/humana) que se liga ao antígeno de superfície CD20 e induz a apoptose dos linfócitos B^5,6^.

Alguns ensaios clínicos sobre glomerulopatias já demonstraram que o RTX é eficaz na vasculite associada a ANCA, na nefropatia membranosa e na crioglobulinemia^7, 8, 9, 10^. Ruggenenti *et al.* publicaram, em 2014, os resultados de sua coorte prospectiva, demonstrando que uma dose de 375 mg/m^2^ foi segura e eficaz na diminuição da dose de esteroides e na redução da recidiva da doença em pacientes que se encontravam em remissão da podocitopatia^11^. Estudos randomizados em pediatria demonstraram que duas a quatro doses de 375 mg/m^2^ por semana prolongam a remissão e reduzem as doses cumulativas de esteroides^12,13^. Nosso estudo tem como objetivo avaliar a eficácia e a segurança do RTX em pacientes com podocitopatias primárias sensíveis ou resistentes a esteroides.

## Métodos

Este é um estudo de coorte retrospectivo de centro único. De janeiro de 1998 a janeiro de 2020, foram analisados os prontuários médicos da clínica ambulatorial de glomerulonefrite da Universidade Federal de São Paulo, Brasil. Incluímos indivíduos que: 1) tinham idade ≥ 12 anos; 2) apresentavam podocitopatia comprovada por biópsia (GESF ou DLM); e 3) receberam pelo menos uma dose de RTX durante um acompanhamento mínimo de 6 meses, prescrita a critério do nefrologista responsável. Esta amostra foi selecionada por processo não aleatório, com base na disponibilidade de dados. Foram excluídos pacientes com proteinúria pré-RTX <0,5 g/24h ou doenças glomerulares secundárias. O protocolo do estudo foi aprovado pelo Comitê de Ética em Pesquisa da Universidade Federal de São Paulo (autorização nº 3.496.678) e elaborado em conformidade com os princípios da Declaração de Helsinque.

Os dados clínicos e laboratoriais utilizados para avaliar os desfechos incluíram proteinúria de 24 horas (ou relação proteína/creatinina, caso não disponível) e albumina sérica em três momentos: até três meses antes da primeira infusão de RTX (T0), 1 a 3 meses após a infusão (T1) e 3 a 6 meses após a infusão (T2). Os sintomas possivelmente associados à infusão de RTX foram registrados nos 12 meses subsequentes. Para estabelecer com maior precisão uma eventual associação entre os sintomas observados e a administração de RTX, também foram registrados os sintomas relatados nos 12 meses anteriores à infusão.

### Desfechos

Foram estabelecidos três critérios de remissão: 1) remissão completa: proteinúria ≤ 0,3 g/24h com TFGe até 25% inferior ao valor de T0; 2) remissão parcial: redução da proteinúria de 24 horas > 50% em relação a T0, proteinúria ≤ 3,5 g/24 h e TFGe até 25% inferior à função renal antes da infusão de RTX; e 3) remissão adaptada: aumento da albumina sérica > 20% ou > 0,5 g/dL em comparação com T0, redução da proteinúria de 24 horas > 35% e TFGe até 25% inferior a T0. A inclusão de um critério alternativo de remissão foi corroborada por achados de estudos anteriores que indicaram um prognóstico favorável mesmo em casos sem critérios clássicos de remissão, particularmente quando acompanhados por aumentos nos níveis séricos de albumina^14, 15, 16, 17^. Esta abordagem baseou-se principalmente no ensaio GEMRITUX, que utilizou um desfecho composto *post hoc*, definido como uma redução da proteinúria superior a 50% em combinação com um aumento da albumina sérica superior a 30%^18^. Também foi avaliada a proporção de pacientes com redução da proteinúria de 24 horas > 35%. Aqueles que não preencheram nenhum dos critérios acima foram considerados refratários ao tratamento.

### Análise Estatística

Os dados foram coletados dos prontuários médicos, organizados no Microsoft Excel^®^ e resumidos por meio de frequências absolutas e relativas (variáveis nominais) e média e desvio padrão (variáveis numéricas). Gráficos de linha foram utilizados para ilustrar as variações nos níveis de proteinúria nos momentos T0, T1 e T2 (Figuras 1 e 2).

**Figura 1:** Evolução da proteinúria de 24 horas no início do estudo (T0), T1 e T2 em pacientes com podocitopatias resistentes a esteroides.

Abreviações – Pt24h: proteinúria de 24 horas.

**Figura 2:** Evolução da proteinúria de 24 horas no início do estudo (T0), T1 e T2 em pacientes com podocitopatias sensíveis a esteroides.

Abreviações – Pt24h: proteinúria de 24 horas.

## Resultados

Foram avaliados 31 pacientes com idade média de 32,9 anos (DP ± 11,0) à infusão de RTX e 24,8 anos (DP ± 13,4) aos primeiros sintomas, dos quais 17 (54,8%) eram do sexo masculino. Dez indivíduos (32,3%) apresentaram os primeiros sintomas antes dos 21 anos e cinco indivíduos (16,1%) antes dos 13 anos. No T0, a TFGe média foi de 74,5 mL/min/1,73 m^2^ (DP ± 32,3), a albumina sérica foi de 3,0 g/dL (DP ± 1,0) e a proteinúria foi de 7,9 g/24h (DP ± 6,2; 74,2% > 3,5 g/24h). Cerca de metade dos participantes (51,6%) eram sensíveis a esteroides e 87,1% receberam outro imunossupressor antes do RTX, especialmente prednisona (35,5%), ciclosporina (19,4%) e ciclosporina e prednisona combinadas (16,1%). A maioria dos pacientes (90,3%) também fazia uso de inibidores do sistema renina-angiotensina-aldosterona (Tabela 1). No T1 (1 a 3 meses após a infusão de RTX), 25,8% alcançaram um dos desfechos do estudo – 23,3% remissão adaptada, 3,2% remissão parcial e 16,1% remissão completa. No T2 (3 a 6 meses após a infusão de RTX), 41,9% atingiram um dos nossos desfechos – 33,3% remissão adaptada, 3,2% remissão parcial e 19,4% remissão completa.

**Tabela 1:** Características basais dos pacientes com podocitopatias tratados com rituximabe

Dados demográficos	N = 31
Sexo masculino, N (%)	17 (54.83)
Idade, anos (DP)	32.90 (11.04)
Idade na ocorrência dos primeiros sintomas, anos (DP)	24.80 (13.42)
**Bioquímica, média (DP)**	
Cr mg/dL	1.34 (0.76)
TFGe ml/min/1,73m^2^	74.52 (32.27)
Proteinúria g/24h	7.95 (6.20)
Albumina sérica g/dL	3.05 (1.00)
**Terapia Pré-Infusão, N (%)**	
Csa + prednisona Csa Prednisona iSRAA Outras Sem ISS	5 (16,13) 6 (19,36) 11 (35,48) 28 (90,32) 5 (16,13) 4 (12,90)
**Características Clínicas**	
Hipertensão, N (%)	21 (67.74)
Dose total de RTX mg/m^2^, média (DP)	663.77 (234.56)
RTX 2 infusões, N (%)	26 (83.87)
Resistente a esteroides, N (%)	15 (48.38)
**Padrão Histológico, N (%)**	
GESF	22 (70.96)
DLM	9 (29.04)

Abreviaturas – Cr: creatinina sérica; Csa: ciclosporina; GESF: glomeruloesclerose segmentar e focal; ISS: imunossupressor; DLM: doença de lesão mínima; iSRAA: inibidor do sistema renina-angiotensina-aldosterona; RTX: rituximabe.

A Figura 3 apresenta a distribuição dos indivíduos de acordo com o status de remissão e a sensibilidade aos esteroides. Entre os pacientes sensíveis aos esteroides, 68,7% apresentaram algum dos desfechos possíveis no T2 – 37,5% remissão completa e 50,0% remissão adaptada. Entre os pacientes resistentes aos esteroides, as taxas de remissão completa e adaptada no T2 foram de 0,0% e 20,0%, respectivamente.

**Figura 3:** Distribuição das remissões completa, completa + parcial, remissão adaptada e redução da proteinúria de 24 horas >35% no T2, de acordo com a sensibilidade aos corticosteroides.

Abreviaturas – RA: Remissão Adaptada; RC: Remissão Completa; RP: Remissão Parcial; Pt 24h > 35%, redução da proteinúria de 24h > 35%.

A Tabela 2 apresenta as características dos indivíduos que não responderam ao RTX (grupo sem remissão) e daqueles que alcançaram: 1) remissão completa ou parcial e 2) remissão adaptada. Considerando apenas as frequências relativas e abstendo-se de análises estatísticas inferenciais devido ao pequeno tamanho da amostra e à seleção não aleatória, observou-se que os pacientes que não responderam receberam uma dose cumulativa menor de rituximabe (643,2 – DP 216,4 vs. 722,8 – DP 301,8), tiveram exposição prévia à ciclosporina (95,6% vs. 37,5%) e apresentaram contagens mais elevadas de células CD19 após a infusão de rituximabe (43,7 – DP 113,9 vs. 3,16 – DP 5,6). Os indivíduos também eram predominantemente hipertensos (65,2%), resistentes a esteroides (65,2%) e apresentaram GESF como padrão histológico (78,3%). Em contraste, os pacientes que alcançaram remissão completa ou parcial após o tratamento com rituximabe apresentaram níveis basais mais elevados de albumina sérica (3,6 – DP 1,0 vs. 2,8 – DP 0,9), sem diagnóstico de hipertensão (12,5%) e eram sensíveis a esteroides (100%). Os pacientes com remissão adaptada apresentaram proteinúria basal mais elevada (12,7 – DP 7,6 vs. 5,7 – DP 4,0), níveis mais baixos de albumina sérica (2,5 – DP 0,9 vs. 3,3 – DP 0,9) e TFGe mais baixa (63,5 - DP 34,7 vs. 80,1 – DP 31,3).

**Tabela 2:** Características clínicas e laboratoriais dos 31 pacientes com podocitopatias de acordo com a presença e o tipo de remissão

	Remissão completa ou parcial		Remissão adaptada	
Características	Remissão (8)	Sem resposta (23)	Remissão (10)	Sem resposta (20)
Idade aos primeiros sintomas, anos (DP)	23.75 (7.70)	25.18 (15.11)	23.44 (12.72)	24.05 (13.06)
Proteinúria pré-infusão g/24h (DP)	6.40 (8.56)	8.50 (5.29)	12.66 (7.60)	5.68 (4.02)
Albumina sérica pré-infusão g/dL (DP)	3.63 (1.04)	2.84 (0.92)	2.46 (0.90)	3.34 (0.93)
TFGe mL/min/1,73m^2^ (DP)	77.52 (42.92)	73.47 (28.80)	63.50 (34.67)	80.14 (31.26)
Dose total de RTX mg/m^2^ (DP)	722.81 (301.85)	643.23 (216.40)	693.03 (280.55)	654.10 (226.76)
Células CD19/mm^3^ (DP), n = 11	3.16 (5.60)	43.72 (113.90)	3.43 (5.42)	50.82 (123.06)
Ciclosporina pré-infusão, N (%)	3 (37.50)	22 (95.65)	8 (80.00)	16 (80.00)
Hipertensão, N (%)	1 (12.50)	15 (65.22)	4 (40.00)	11 (55.00)
GESF, N (%)	4 (50.00)	18 (78.26)	9 (90.00)	13 (65.00)
DLM, N (%)	4 (50.00)	5 (21.74)	1 (10.00)	7 (35.00)
Sensível a esteroides, N (%)	8 (100)	8 (34.78)	7 (70.00)	9 (45.00)

Abreviaturas – TFGe: Taxa de Filtração Glomerular Estimada; DLM: Doença de Lesão Mínima; GESF: Glomeruloesclerose Segmentar e Focal; RTX: Rituximabe.

A Tabela 3 apresenta os sintomas autorrelatados no período de 1 ano antes e após o RTX. Até 12 meses após a infusão de RTX, a maioria dos indivíduos não relatou eventos adversos (61,3%), seguidos por exantema (9,7%), artralgia (6,5%) e pneumonia (6,5%). Não houve aumento na ocorrência de eventos adversos após a infusão de RTX em comparação aos 12 meses anteriores ao tratamento. Não foram registrados óbitos e um dos pacientes evoluiu para DRCVD em 12 meses (um paciente de 22 anos, do sexo masculino, que consultou um nefrologista pela primeira vez um ano antes da primeira infusão de RTX devido a uma GESF resistente a esteroides).

**Table 3:** Efeitos adversos autorrelatados até 1 ano antes e após a infusão de rituximabe

Eventos adversos	Antes, N	%	Após, N	%
Infecção do trato urinário	4	12.90%	1	3.23%
Artralgia	4	12.90%	2	6.45%
Infecção viral	3	9.68%	1	3.23%
Infecção cutânea	2	6.45%	0	0%
Pneumonia	2	6.45%	2	6.45%
Exantema	0	0%	3	9.68%
Outros	5	16.13%	4	12.90%
Nenhum	13	41.94%	19	61.30%

Os gráficos de linha (Figuras 1 e 2) ilustram a progressão da proteinúria após a administração de RTX nos momentos T0, T1 e T2, sugerindo que as reduções na proteinúria foram mais significativas e ocorreram com maior frequência em indivíduos sensíveis a esteroides em comparação aos resistentes, embora também seja possível observar uma redução da proteinúria em alguns casos resistentes a esteroides.

## Discussão

Esta coorte retrospectiva teve como objetivo avaliar a eficácia e a segurança do RTX de acordo com os registros médicos de 31 pacientes com podocitopatia primária comprovada por biópsia. Além disso, foram exploradas algumas características clínicas que podem influenciar a remissão completa e parcial.

São urgentemente necessárias novas e melhores opções para o tratamento das podocitopatias, uma vez que pacientes com GESF apresentam uma taxa de remissão de quase 50% e um risco de progressão para DRCVD de até 20% em um período de dez anos. Além disso, esses indivíduos estão expostos a doses cumulativas substanciais de esteroides ao longo da vida, o que leva a um risco significativo de eventos adversos^19, 20, 21^. O RTX já foi sugerido como eficaz em outras glomerulopatias, como nefropatia membranosa e vasculite associada a ANCA, mas ainda faltam evidências sólidas de eficácia em podocitopatias^7, 8, 9, 10,22^.

Nossa coorte apresentou taxas de remissão inferiores às relatadas por outros autores, possivelmente porque aproximadamente metade de nossos pacientes (48,4%) era resistente a esteroides. Uma metanálise de 2020 demonstrou uma taxa de remissão (parcial e completa) de 73,7%; no entanto, a maioria dos estudos se concentrou em pacientes sensíveis a esteroides. Ao considerar somente tais pacientes, 50,0% alcançaram remissão parcial ou completa após o uso de RTX, em consonância com os achados de Hansrivijit *et al*.^23^.

Embora a dose de RTX não tenha se associado à remissão clínica, Chan *et al.*, em uma coorte retrospectiva, observaram taxas mais elevadas de recidiva em 511 crianças que utilizaram uma dose total de 375 mg/m^2^ em comparação com > 750 mg/m^2 24^. Por outro lado, Ruggenenti *et al.*, em 2014, observaram que 375 mg/m^2^ foi altamente eficaz na prevenção das recidivas em pacientes com GESF ou DLM em remissão^11^.

Como um anticorpo, o RTX pode ser excretado na urina de pacientes com síndrome nefrótica, sendo tradicionalmente recomendado seu uso após a indução da remissão em podocitopatias. É importante notar que a maioria dos nossos pacientes apresentava síndrome nefrótica no T0, mas, ainda assim, observamos uma taxa de remissão razoável ao considerar os pacientes sensíveis a esteroides, utilizando uma dose média total de RTX de 663,7 mg/m^2^. Os pacientes que não responderam receberam, em média, doses menores de rituximabe, embora a ausência de análise inferencial impeça conclusões mais sólidas. A falta de ensaios randomizados em adultos dificulta recomendações de uma posologia específica em podocitopatias, mas a responsividade aos esteroides, o estado de remissão e o padrão histológico devem ser considerados antes da definição da dose de RTX^24, 25, 26, 27^.

A hipertensão e o uso prévio de ciclosporina foram associados à ausência de resposta ao RTX, provavelmente refletindo uma doença mais refratária. O padrão histológico da GESF também foi um fator de risco para a ausência de resposta, com apenas 18,2% (4 de 22 pacientes) apresentando remissão parcial ou completa, enquanto 44,4% dos pacientes com DLM alcançaram a remissão. O mesmo foi observado por outros grupos, conforme demonstrado por Hansriviji *et al.*, que encontraram taxas de remissão parcial e completa de 80,3% na DLM e 53,6% na GESF^23^.

Embora os subtipos de GESF – primária, genética e secundária – compartilhem características clínicas como proteinúria nefrótica, eles diferem em sua fisiopatologia e, em geral, não podem ser distinguidos pela histologia^28,29^. Essas diferenças podem influenciar as taxas de resposta, com mecanismos não imunológicos potencialmente mais prevalentes no grupo resistente a esteroides. Curiosamente, cinco pacientes resistentes a esteroides (33,3%) apresentaram uma redução > 35% na proteinúria, possivelmente por um efeito não mediado pelo sistema imunológico. Além de seu efeito bem conhecido sobre as células B, o RTX estabiliza o citoesqueleto dos podócitos por meio da regulação da esfingomielinase ácida e da conexão com a esfingomielinase ácida-similar-3b, ambas as quais podem reduzir a proteinúria^30,31^. Este fato pode explicar por que 20,0% dos pacientes no grupo resistente a esteroides apresentaram remissão adaptada.

O papel do RTX nas podocitopatias resistentes aos esteroides permanece incerto. Em nossa coorte, nenhum dos quinze pacientes resistentes aos esteroides apresentou remissão completa ou parcial, mas 3 (20,0%) apresentaram remissão adaptada e 5 (33,3%) uma redução superior a 35% na proteinúria de 24 horas, com uma dose relativamente elevada de RTX. Um estudo não randomizado e não controlado, realizado na *Mayo Clinic* com 9 pacientes, não demonstrou significância estatística na redução da proteinúria após seis meses (5,9 ± 4,6 g/24h) e após 12 meses (7,7 ± 4,6 vs. 7,2 ± 7,3 g/24h)^32^. Por outro lado, uma revisão sistemática incluindo 226 crianças com síndrome nefrótica resistente a esteroides mostrou uma taxa de resposta de 39,2% na GESF^33^. Considerando a heterogeneidade das evidências, as diretrizes do KDIGO de 2021 para glomerulopatias colocam o RTX como opção terapêutica apenas em pacientes sensíveis a esteroides, mas enfatizam a necessidade de estudos adicionais de alto impacto para uma resposta definitiva^22^.

Também observamos uma menor prevalência de eventos adversos (infecções, 12,9%; exantema, 9,7%; artralgia, 6,5%) em nossa coorte em comparação com outros estudos, um achado que pode ter sido influenciado por uma menor taxa de RTX em combinação com outros imunossupressores. Em uma coorte retrospectiva de 468 pacientes com idade ≥ 21 anos, os principais eventos adversos observados até 24 meses após a infusão de RTX foram as infecções (47,9%), das quais parte foi considerada grave (37,5%). De forma semelhante, hipogamaglobulinemia de IgM foi identificada em 40,8% dos pacientes, 19,1% apresentaram anafilaxia e 19,0% apresentaram reações à infusão – hipotensão (3,4%), sintomas respiratórios (3,0%), tremores (3,0%) e exantema ou prurido (2,6%)^34^. Em outro estudo envolvendo 2.578 pacientes com artrite reumatoide, observou-se uma taxa de reação à infusão de 25%; no entanto, apenas 1% dessas reações foram classificadas como graves^35^.

Como um anticorpo quimérico, o RTX pode induzir reações relacionadas à infusão. Dada a natureza retrospectiva do nosso estudo, é possível que sintomas leves tenham sido suprimidos, o que explicaria a baixa incidência de reações relacionadas à infusão. Por outro lado, a ausência de documentação sugere que os eventos não registrados provavelmente foram de natureza leve.

Os pontos fortes deste estudo incluem: 1) trata-se de uma das maiores coortes envolvendo o uso de RTX em podocitopatias; 2) oferece novos *insights* ao sugerir que os níveis séricos de albumina e proteinúria pré-infusão, além da hipertensão e da responsividade aos esteroides, podem predizer a resposta clínica ao RTX; 3) demonstra redução da proteinúria em alguns pacientes resistentes aos esteroides, corroborando a hipótese de que o RTX possa exercer algum efeito nesses pacientes; 4) a maioria dos nossos pacientes apresentou síndrome nefrótica no T0, desafiando a crença comum de que o RTX deva ser utilizado durante a remissão para minimizar perdas urinárias.

Algumas das limitações do nosso estudo são: 1) seu desenho retrospectivo é afetado pelo viés de memória nos sintomas autorreferidos e os dados estão restritos aos registros médicos; 2) a pequena dimensão da amostra, impossibilitando uma análise inferencial; 3) diferentes regimes de infusão de RTX, o que leva a doses e intervalos não padronizados; 4) uso concomitante de outros medicamentos imunossupressores, dificultando a atribuição dos efeitos observados exclusivamente ao RTX; 5) ausência de testes genéticos, para uma melhor definição dos subtipos de GESF.

Em conclusão, nossos resultados sugerem que o RTX constitui uma opção terapêutica útil não apenas para podocitopatias sensíveis a esteroides, mas também em casos selecionados de resistência a esteroides, determinando uma redução da proteinúria que pode contribuir positivamente para o manejo clínico dessas doenças glomerulares. O RTX mostrou-se geralmente eficaz e bem tolerado nesta coorte de pacientes adultos.

## Data Availability

Os dados subjacentes a este artigo serão compartilhados mediante solicitação razoável ao autor correspondente.
